# Identified *S100A9* as a target for diagnosis and treatment of ulcerative colitis by bioinformatics analysis

**DOI:** 10.1038/s41598-024-55944-3

**Published:** 2024-03-06

**Authors:** Lulu Tan, Xin Li, Hong Qin, Qingqing Zhang, Jinfeng Wang, Tao Chen, Chengwu Zhang, Xiaoying Zhang, Yuyan Tan

**Affiliations:** 1 The First College of Clinical Medical Science, China Three Gorges University and Yichang Central People’ Hospital, Yichang, 443000 China; 2 Wuhan Asia Heart Hospital, Wuhan, 430022 China; 3 Haiyan County Hospital of Traditional Chinese Medicine, Jiaxing, 314399 China

**Keywords:** Ulcerative colitis, Bioinformatics analysis, Immune infiltration, Diagnostic biomarkers, *S100A9*, Computational biology and bioinformatics, Immunology, Molecular biology, Biomarkers

## Abstract

Ulcerative colitis (UC) is a chronic, recurrent inflammatory bowel disease. UC confronts with severe challenges including the unclear pathogenesis and lack of specific diagnostic markers, demanding for identifying predictive biomarkers for UC diagnosis and treatment. We perform immune infiltration and weighted gene co-expression network analysis on gene expression profiles of active UC, inactive UC, and normal controls to identify UC related immune cell and hub genes. Neutrophils, M1 macrophages, activated dendritic cells, and activated mast cells are significantly enriched in active UC. *MMP-9, CHI3L1, CXCL9, CXCL10, CXCR2* and *S100A9* are identified as hub genes in active UC. Specifically, *S100A9* is significantly overexpressed in mice with colitis. The receiver operating characteristic curve demonstrates the excellent performance of *S100A9* expression in diagnosing active UC. Inhibition of *S100A9* expression reduces DSS-induced colonic inflammation. These identified biomarkers associated with activity in UC patients enlighten the new insights of UC diagnosis and treatment.

## Introduction

Ulcerative colitis (UC) is a chronic and recurrent inflammatory bowel disease (IBD) with increasing incidence worldwide^1^. UC confronts with severe challenges including the unclear pathogenesis and lack of specific diagnostic markers, demanding for identifying predictive biomarkers for UC diagnosis and treatment.

Immune disorders play a crucial role in the etiology of UC among the complex pathogenic mechanisms^2,3^. Infiltrating immune cells in the intestinal mucosa of UC patients leads to mucosal inflammation. Early evidence suggests that UC is driven by Th2-polarized T cells in the lamina propria of the colon^4^. Additionally, neutrophils are key mediators of epithelial cytotoxicity and barrier dysfunction in UC^5^. Recently, targeting immune cells to inhibit inflammation has become a research hotspot. For example, 260 Itaconate modified cysteine sites were found in the macrophage proteome, and Itaconate could covalently modify the cysteine of macrophages to play an anti-inflammatory role^6^. However, the proportion and composition of infiltrating immune cells in the intestinal mucosal tissue of active UC remain unclear. Therefore, revealing the immune cells closely related to the pathogenesis of UC may provide a new direction for its treatment.

Limited achievements have been made in the field of diagnosis and surveillance of UC. The diagnosis of UC mainly relies on invasive tests including colonoscopy and histopathology. Besides, colonoscopy is highly costed and associated with complications such as perforation^7,8^. Although identified markers such as fecal calprotectin, C-reactive protein, and Intercellular adhesion molecule 1 were associated with disease activity, they were inaccurately correlated with endoscopic status^9,10^. Thus, it is emergently demanded for newly noninvasive and economic strategies for UC diagnosis.

Microrray technology have provided important insights into the pathophysiology mechanisms of disease at the genetic level^11,12^. Bioinformatics analysis has been widely used to search for differentially expression genes (DEGs), miRNA, and functional pathways involved in the development and progression of UC. For example, Wu et al. constructed a complete lncRNA-miRNA-mRNA network through bioinformatics to determine the specific immune infiltration characteristics of UC^13^. Moreover, protein–protein interaction (PPI) can provide information about direct and indirect protein interactions for screening hub genes^14,15^.

This study explored the difference of immune cell infiltration of UC in normal intestinal mucosal tissues through biological information. The hub genes (*CXCL9, CXCL10, MMP-9, CHI3L1, CXCR2* and *S100A9*) of UC were identified by DEGs, weighted gene co-expression network analysis (WGCNA) and protein–protein interaction (PPI) network analysis. Then, we verified the results of bioinformatics analysis using external cohort and in vitro experiments, and explored the mechanism of hub genes in UC. Comprehensive analysis of immune infiltrating cells and hub genes will provide new biomarkers for the diagnosis and treatment of UC.

## Materials and methods

### Microarray data

The gene expression profile of GSE87466 was obtained from the GEO database (http://www.ncbi.nlm.nih.gov/geo/). A total of 108 mucosal biopsy samples were obtained from 87 active UC patients and 21 control subjects for subsequent analysis. The platform for GSE107499 was based on the GPL15207 Affymetrix Human Gene Expression Array, containing 75 active and 44 inactive UC mucosal biopsy samples.

### Data preprocessing and DEGs analysis

All raw data were normalized and standardized by using the R software package. Gene differential expression analysis was conducted through the “limma” packages in the Bioconductor package (available online: http://www.bioconductor.org/). *p* value < 0.05 and |log_2_FC|> 2 were set as cut-off standards and considered to indicate statistical significance.

### Gene set enrichment analysis (GSEA) and gene set variation analysis (GSVA)

In the training sets GSE87466 and GSE107499, a chip expression profile file and a sample data file (UC vs control, active vs inactive), respectively, were created and imported into the GSEA software^16^. Hallmark gene sets were selected to obtain pathway enrichment results for total gene expression levels. NOM *p*-value < 0.05 and FDR q-value < 25% were considered significantly enriched. To explore the biological function of *S100A9* in UC, GSVA was performed on *S100A9* high expression group and *S100A9* low expression group^17^.

### Immune cell infiltration

Gene expression datasets for GSE87466 and GSE107499 were uploaded to the CIBERSORT portal (http://cibersort.stanford.edu/) in the accepted CIBERSORT format. The original CIBERSORT gene signature file LM22, which defines 22 immune cell subtypes, was used to analyze immune cell infiltration in UC tissues and normal tissues, active UC tissues and inactive UC tissues^18^. The samples were screened according to *P* value < 0.05.

### WGCNA analysis

The top 25% of genes with the largest variance in the gene expression dataset of GSE87466 were extracted to perform WGCNA. The R package “WGCNA” was applied to find clinical traits-related modules. In order to ensure the reliable results of network construction, one outlier sample was removed. Here, soft-thresholding power was set to 12 to convert the similarity matrix of gene expression into an adjacency matrix. The fitting degree of scale-free topological model was 0.80. Then, the adjacency relationship was transformed into a topological overlap matrix. The dynamic tree cutting method was used for module clustering, merging closer modules into new modules with a height of 0.2. Each gene network module sets a minimum number of bases of 50.

### Gene ontology and KEGG pathway enrichment analysis

DAVID (https://david.ncifcrf.gov) is an online bioinformatics tool designed to identify a large number of gene or protein functions^19^. DAVID software was used to perform GO (including Biological process, cellular component and molecular function) and KEGG pathway analysis^20^. *p* value < 0.05 was considered to indicate statistical significance.

### Protein–protein interaction network and hub gene definition

To predict protein–protein interactions, the online databases STRING^21^ and GENEMANIA^22^ were utilized in the PPI network analysis. The integrated regulatory networks were then visualized by cytoscape. The degree of protein nodes was calculated by using the cytoscape plugin, cytohubba, to find top10 hub genes.

### Data validation

To verify the robustness of hub genes, the microarray data of GSE59071 was obtained from GPL6244 [HuGene-1_0-st] Affymetrix Human Gene 1.0 ST Array and included 97 colonic mucosal tissues from patients with active UC and 11 healthy colon mucosal tissues. The microarray data of GSE126124 [HuGene-1_0-st] Affymetrix Human Gene 1.0 ST Array [transcript (gene) version], which included 57 peripheral blood samples (18 UC samples and 39 control samples), were downloaded from the GEO database.

### Animals and experimental design

SPF grade healthy female C57BL/6 mice aged 6–8 weeks were purchased from Beijing SiPeiFu Bio-Technology. Acute colitis was induced in mice by continuous feeding with 3% dextran sulfate sodium (DSS) (MPbio, USA) for 8 days. Mice were anesthetized by intraperitoneal injection of pentobarbital sodium. During the experiment, the body weight, fecal characteristics and blood in the stool of each mouse were recorded every day. According to the instructions, the mice in the treatment group were intraperitoneally injected with paquinimod (MedChemExpress, ABR 25757) at 5 mg/kg every 2 days. The mice were sacrificed (cervical disloaction) humanely and the colon length of each group of mice was measured after the experiment. Body weight, colon length and hematoxylin–eosin (H&E) staining were used to evaluate the severity of colitis in each group of mice. All animal procedures were performed in accordance with the Three Gorges University Institutional Animal Care and Use Committee.

### Quantitative real-time PCR

According to the manufacturer’s instruction, total RNA from UC groups and controls colon tissues was extracted using a Trizol reagent kit (Takara, Dalian, China). qRT-PCR was then performed as described previously^23^. All primers were synthesized by TSINGKE (Shanghai, China) (Table S1). *β-Actin* was used as an internal control.

### Western blotting

Tissues protein extraction was carried out on ice using a RIPA buffer (Sigma-Aldrich, Darmstadt, Germany) containing proteinase and phosphatase inhibitors. Western blotting was performed as described previously^23^. Primary antibodies against *S100A9* (Abcam, ab242945, Cambridge, UK, 1:1000 dilution), and *β-actin* (Proteintech, 66009-1-Ig, Wuhan, China, 1:5000 dilution). *β-actin* was used to normalize the protein level.

### Immunohistochemistry

Immunohistochemical analysis of mouse intestinal tissue was performed using anti-*S100A9* (Abcam, ab242945, Cambridge, UK, 1:200 dilution), anti-*MPO* (Abcam, ab208670, Cambridge, UK, 1:1000 dilution), or anti-*F4/80* (CST, #70076, Danvers, USA, 1:1000 dilution) antibodies and incubated overnight at 4 °C. Immunohistochemical was performed as described previously^23^.

### Statistical analysis

Data was analyzed using the R software. GraphPad prism 8.00 software was used to calculate the area under the curve. Statistical significance between the two groups was calculated by Student's *t*-test. ****P < 0.0001; ***P < 0.001; **P < 0.01; *P < 0.05; ns, not significant.

### Ethical approval and informed consent

The China Three Gorges University Ethics Committee gave its approval to the animal experimentation methodology. The study is reported in accordance with ARRIVE guidelines.

## Results

### Immune infiltration in active UC

**Figure 1:**
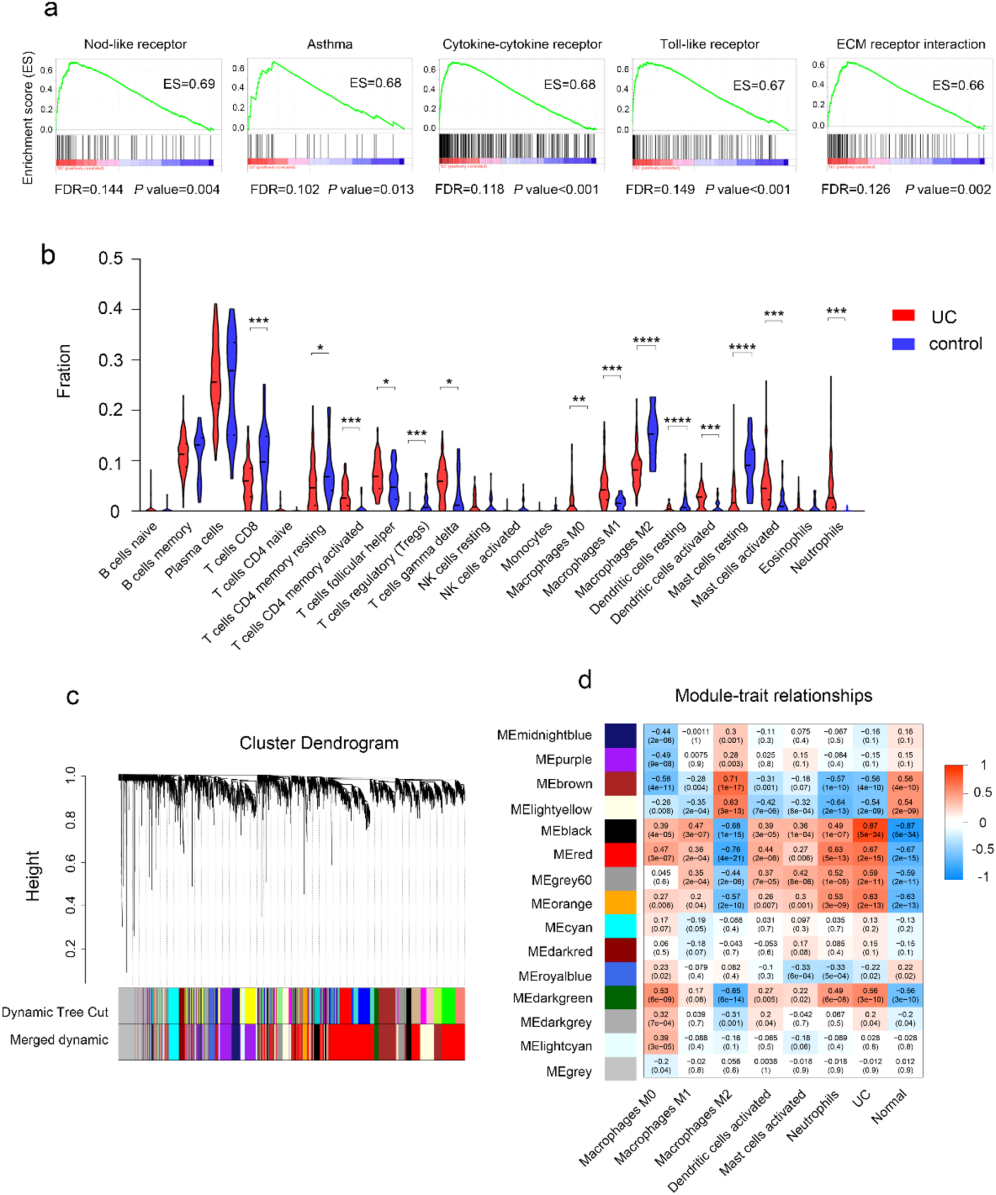
Immune infiltration in active UC. (**a**) GSEA of KEGG pathways in active UC and normal tissues. (**b**) Violin plot comparing the proportions of 22 types of immune cells between active UC and normal tissues. (**c**) Merge similar modules. (**d**) Heatmap of the correlation between the modules and UC.

To understand the whole-gene enrichment annotation of UC and consider the potential role of genes with smaller differences in expression in UC and normal tissues, GSEA was conducted to search KEGG pathways enriched in the active UC. GSEA results showed that active UC was mainly enriched in nod-like receptors, cytokine-cytokine receptor interactions, Toll-like receptors, ECM receptor interactions, cell adhesion molecules, chemokines, leukocyte transendothelial migration and other inflammatory signaling pathways (Fig. 1a, S1a). To further elucidate the immune infiltration of active UC and normal tissues, CIBERSORT algorithm was used to calculate the proportion of 22 immune cell subsets between 87 UC and 21 normal tissues (Fig. S1b), 75 active UC and 44 inactive UC samples (Fig. S1c). M0 macrophages, M1 macrophages, activated DCs, activated mast cells, neutrophils, activated CD4^+^ memory T‐cells and gamma delta T‐cells were upregulated in active UC. Regulatory T cells, M2 macrophages, resting DCs, resting mast cells were downregulated in active UC (Fig. 1b, S1d). Correlation analysis suggests that the functions of activated mast cells, M1 macrophages, and follicle-assisted T cells may promote each other, while M2 macrophages may antagonize M1 macrophages and activated mast cells (Fig. S2a,b).

According to the corresponding steps of WGCNA described in the materials and methods, 15 modules were obtained after merging similar genes (Fig. 1c, S3a–c). According to the correlation diagram between modules and clinical information, the correlation between black modules and active UC was the highest, with a correlation coefficient of 0.87 (*p* = 5e−34). Followed by the red module (r = 0.67, *p* = 2e−15). Meanwhile, the black module and the red module are positively correlated with the expression of neutrophils, M1 macrophages, and negatively correlated with the M2 macrophages (Fig. 1d). Therefore, the black and red modules were considered the most study-worthy.

### Identification of DEGs and functional enrichment

**Figure 2:**
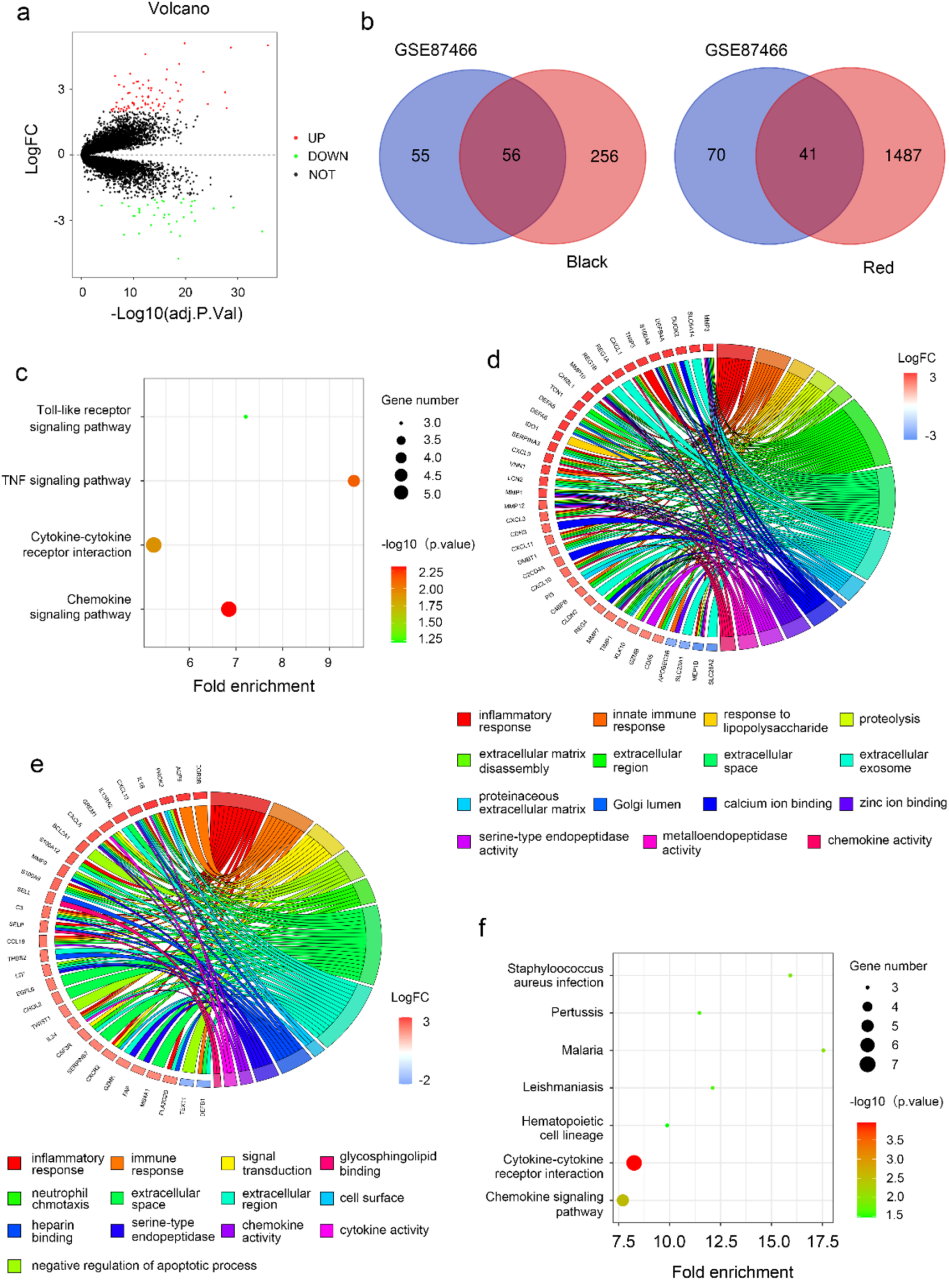
Identification of DEGs and functional enrichment. (**a**) The volcano plot of DEGs in UC and normal tissues. (**b**) Venn diagram of DEGs and module related genes. (**c**) KEGG signal pathway enrichment analysis diagram of differentially expressed black module related genes. (**d**) GO enrichment analysis diagram of differentially expressed black module related genes. (**e**) GO enrichment analysis diagram of differentially expressed red module related genes. (**f**) KEGG signal pathway enrichment analysis diagram of differentially expressed red module related genes.

In order to explore the differential gene expression between active UC and normal intestinal mucosal tissues, “Limma” package was used to identify DEGs in GSE87466. A total of 111 DEGs were identified between the UC group and the normal control group, including 37 downregulated genes and 74 upregulated genes (Fig. 2a). The Venn diagram showed the overlap between the DEGs and the black and red modules, respectively (Fig. 2b). Additionally, in related to UC activity, the black and red modules were assessed for further functional enrichment, consisting of GO enrichment and KEGG pathway analysis of module genes of interest. KEGG pathway analysis of DEGs in black and red module demonstrate that the most significant pathways are cytokine-cytokine receptor interaction, chemokine signaling pathway and TNF signaling pathway (Fig. 2c,f). The results of the GO analysis of DEGs in black and red module were shown in Fig. 2d and e. The DEGs were particularly enriched in inflammatory response, extracellular space and chemokine activity.

### Identification of hub genes

**Figure 3:**
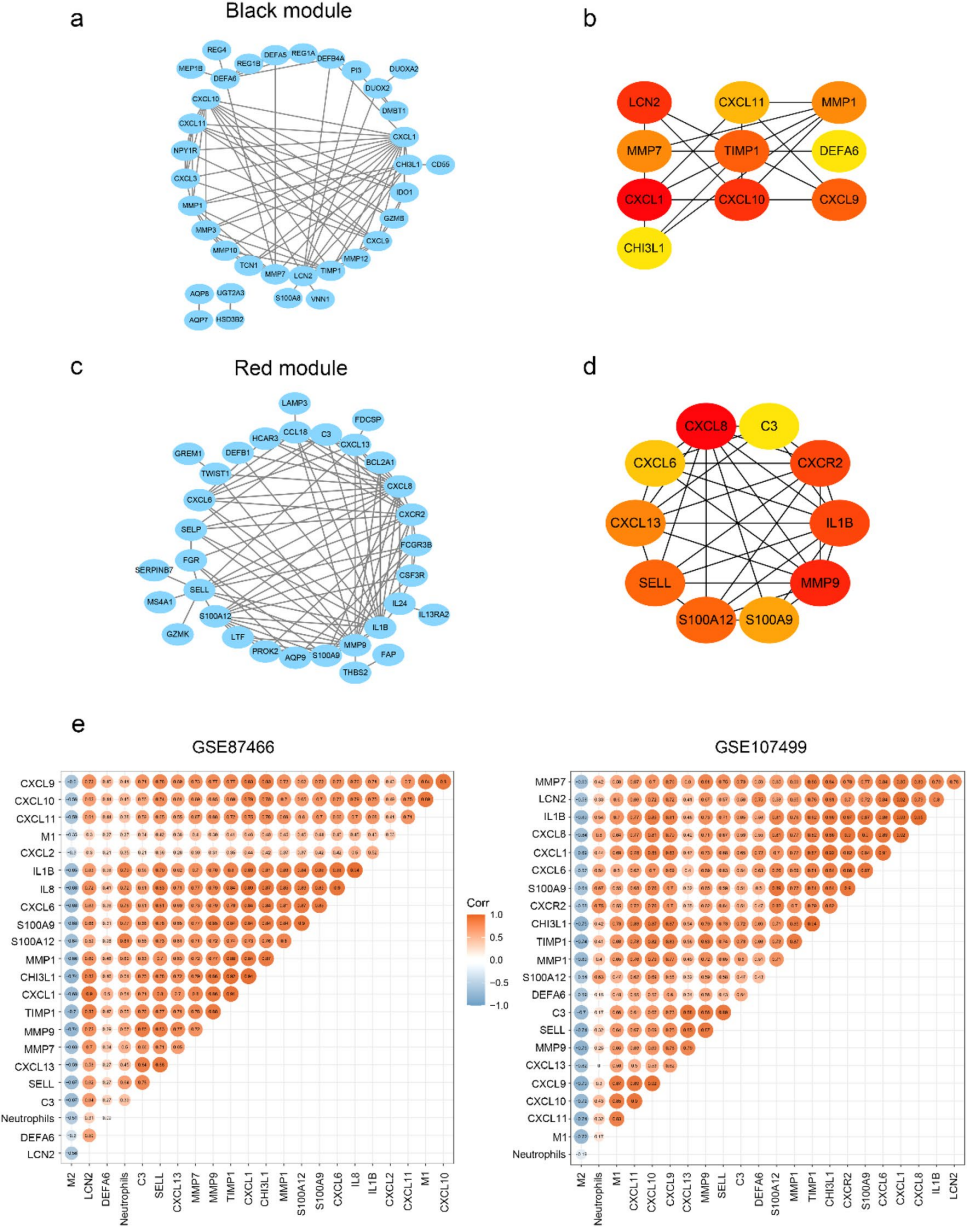
Visualization and module identification of the PPI network. PPI network for DEGs in the black (**a**) and red (**c**) modules. Top 10 core targets of the black (**b**) and red (**d**) module in the PPI network. (**e**) Pearson correlation analysis of the corresponding hub genes.

To identify candidate biomarkers related to UC activity, the STRING and GENEMANIA database were applied to identify the interactions between DEGs in black and red module (Fig. 3a,c, Fig. S4a,b). Cytoscape was then used to construct a network of PPI for DEGs in the black and red modules, respectively (Fig. 3a,c). Top 10 hub genes in the black and red module were identified in PPI network respectively (Fig. 3b,d). “Corrplot” R package was used to further calculate the relationship between hub genes and three types of immune cells (M1 macrophages, M2 macrophages and neutrophils). The results showed that *MMP-9* and *CHI3L1* had the most negative correlation with M2 macrophages, *CXCL9* and *CXCL10* exhibited the most positive correlation with M1 macrophages, *S100A9* and *CXCR2* were most positively correlated with neutrophils (Fig. 3e).

### Data validation

**Figure 4:**
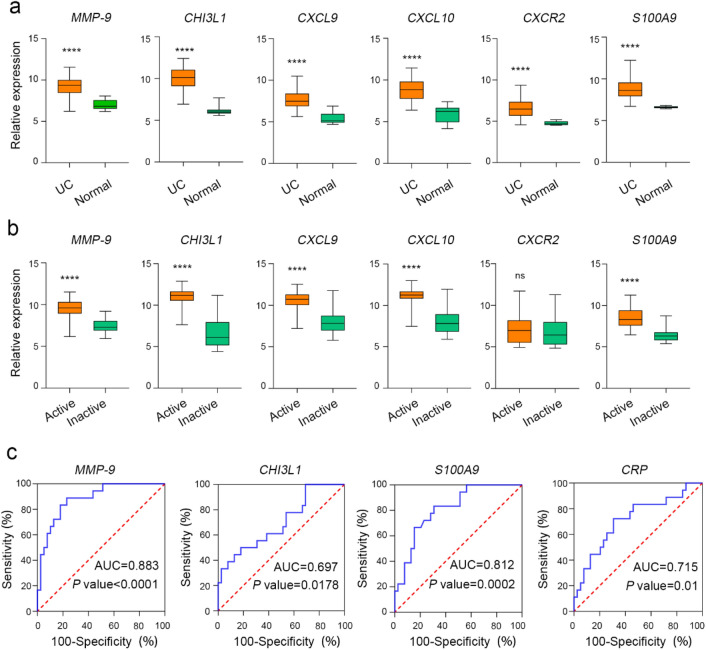
Validation of hub genes. (**a**) Relative expression of six hub genes in UC versus healthy patients. (**b**) Relative expression of six hub genes in active UC versus inactive UC. (**c**) ROC curve of *CRP, MMP-9, S100A9 and CHI3L1*.

To verify the reliability and robustness of the above six genes, two independent datasets were used for external validation. Compared with healthy and inactive UC patients, the expression of *MMP-9, CHI3L1, CXCL9, CXCL10*, and *S100A9* were significantly increased in active UC patients (Fig. 4a,b). Transcriptome expression profiles of whole blood samples from UC and paired patients were used to identify potential molecules for UC diagnosis. The diagnostic accuracy of *MMP-9* and *S100A9* was 0.883 and 0.812, respectively (Fig. 4c), which was significantly higher than that of *CRP*, a commonly used clinical indicator.

### S100a9 is highly expressed in intestinal inflammatory mucosal tissue

**Figure 5:**
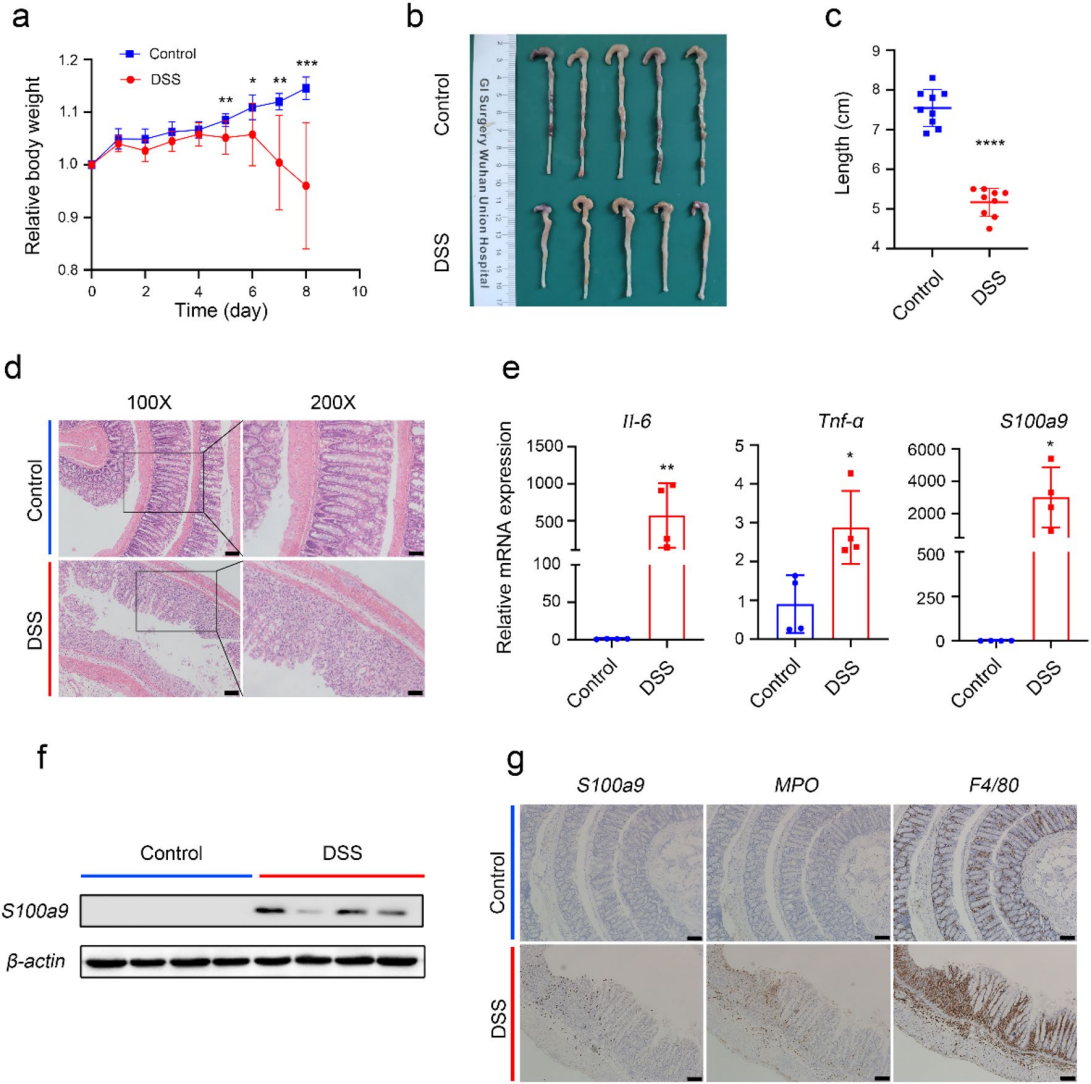
*S100a9* is highly expressed in intestinal inflammatory mucosal tissue. DSS-induced colitis in C57BL/6 mice was induced as indicated. (**a**) Relative body weight. (**b**) Gross macroscopic architecture of colonic tissues on day 8. (**c**) Colon length. (**d**) Colonic sections were stained with H&E (H&E staining, left panels: original magnification 100×, scale bar: 100 µm; right panels: original magnification 200×, scale bar: 50 µm). (**e**) QRT-PCR analysis of *Il-6, Tnf-α* and *S100a9* mRNA levels. (**f**) Western blotting of *S100a9*. Original blots/gels are presented in Supplementary Fig. S7. (**g**) Immunohistochemical (IHC) analysis of *S100a9*, *MPO* and *F4/80*, scale bar: 100 µm.

In order to verify whether in vivo experiments are consistent with our bioinformatics analysis results, the colitis model was carried out. Compared with the control group, the body weight and colon length of mice in DSS treatment group were significantly reduced (Fig. 5a–c). Histological lesions and inflammatory cytokine (*Il-6, Tnf-α*) levels indicate that the colitis model is successfully established (Fig. 5d,e). The mRNA expression levels of *Cxcr2, Chi3l1* and *S100a9* in colitis mice were significantly higher than those in control group, especially *S100a9*, which was consistent with our bioinformatics analysis results (Fig. 5e, S5a). Finally, *S100a9* was selected from the six hub genes for further analysis. Consistent with the results of the qPCR experiment, the *S100a9* protein was overexpressed in the intestinal tissue of colitis mice (Fig. 5f, S7). Histochemical results showed that the expression of *S100a9* in intestinal tissue of colitis mice was increased, and it was positively correlated with neutrophils and macrophages (Fig. 5g). Furthermore, the gene set variation analysis results demonstrate that *IL‐2/STAT5, IL‐6/JAK/STAT3* signalling, apoptosis, angiogenesis and epithelial‐mesenchymal transition (EMT) are highly enriched in active UC with high *S100a9* expression (Fig. S5b).

### Blockade of S100a9 alleviates DSS-induced colitis in mice

**Figure 6:**
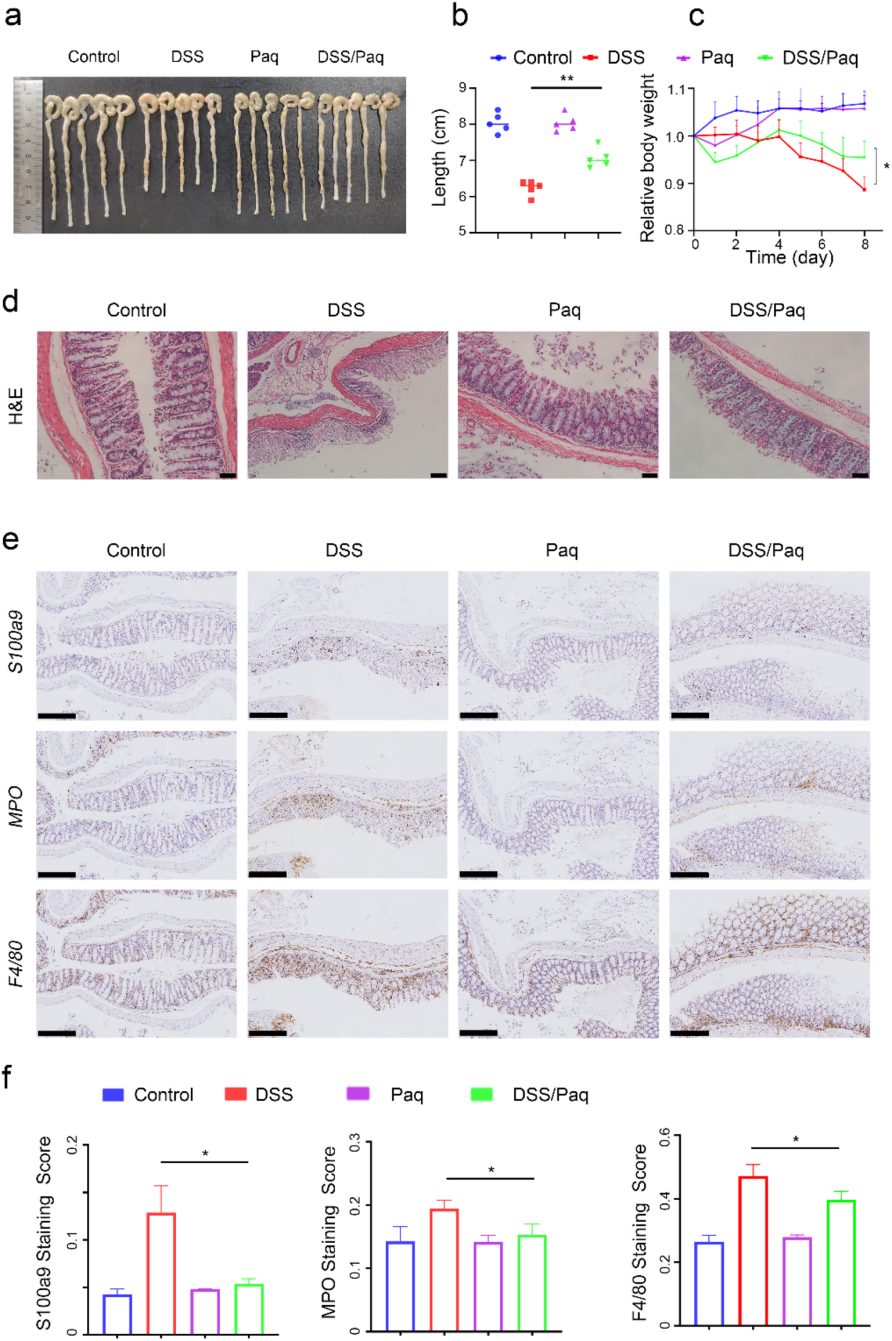
Blockade of *S100a9* alleviates DSS-induced colitis in mice. Two groups of DSS-exposed mice (*n* = 5) were treated with paquinimod (5 mg/kg) or PBS as controls every two days intraperitoneally. Two groups of none DSS-exposed mice (*n* = 5) were also treated with paquinimod (5 mg/kg) or PBS as negative controls. (**a**) Gross macroscopic architecture of colonic tissues. (**b**) Colon length. (**c**) Relative body weight. (**d**) H&E staining analysis of colon tissues, scale bar: 50 µm. (**e**) IHC analysis of *S100a9*, *MPO* and *F4/80*, scale bar: 250 µm. (**f**) IHC staining score.

To determine the role of *S100a9* in the pathogenesis of colonic inflammation, the *S100a9*-selective inhibitor paquinimod (Paq, 5 mg/kg) was administered intraperitoneally every two days. Paq had no obvious effect on mouse growth (Fig. 6a,c). Compared with DSS treated mice, Paq-treated mice had less symptoms of colitis/ weight loss/ intestinal mucosal destruction and less shortening colon (Fig. 6a–d, S6). Meanwhile, the intestinal tissue of the Paq-treated mice showed less immune cell (neutrophils and macrophages) infiltration (Fig. 6e,f). Taken together, our data suggest that *S100a9* may serve as a potential therapeutic target for UC.

## Discussion

In this study, we performed immune infiltration analysis on UC (active UC and inactive UC) patients and healthy volunteers, and find that a variety of immune cells such as neutrophils, macrophages and activated DCs were highly enriched in active UC. Among these immune cells, neutrophils, M1 macrophages and DCs can participate in the proinflammatory response in a variety of ways, such as producing granular lyase and neutrophils extracellular traps, and secreting pro-inflammatory cytokines including *IL-6, IL-1β and TNF-α*^24–27^. In contrast, M2 macrophages produces a large number of anti-inflammatory cytokines (IL-10) and anti-inflammatory chemokines (*CCL-17, CCL-24*) that are involved in suppressing immune responses and tissue healing^28^. To date, there are few reports on the involvement of mast cells in UC. Here we showed that activated mast cells increased significantly in UC patients, while resting mast cells decreased, suggesting that mast cells may be involved in the pathogenesis of UC. In addition, no significant changes in NK cells were observed in our study, suggesting that NK cells may not be involved in the pathogenesis of UC. Regulatory cells (Tregs) play an important role in suppressing the immune response and maintaining peripheral tolerance^29^. We found that the number of Tregs increased in UC patients compared with healthy individuals, which is inconsistent with the results of Yao et al.^30^. One possible reason is that the expression level of Tregs are related to the disease progression of UC. Also, we observed a significant increase of memory B cells in the intestinal tissues of patients with active UC, but the function of memory B cells in the pathogenesis of UC remains to be studied. It has been reported that circulating memory B cells are associated with serum immunoglobulin level in patients with ulcerative colitis and may be involved in the pathogenesis of UC^31^.

We screened the genes most related to UC through WGCNA analysis, and obtained two modules, which were also significantly correlated with the differentially expressed immune cells (neutrophils, macrophages and activated DCs). In addition to inflammatory responses and innate immune responses, we also found that differential genes in the modules were particularly enriched in multiple metabolic pathways, such as extracellular space, serine endopeptidase activity, and zinc ion binding. There is increasing evidence showing that the extracellular space is important for UC initiation^32^. According to free radical induction theory, excessive hydrogen peroxide can be produced by abnormal metabolism of colonic epithelial cells, which can cause extensive oxidative damage to the intestinal barrier^33^. Recently, serine endopeptidase activity was reported to be most prominent among several protein functions associated with UC^34^. Besides, overexpression of zinc-binding protein may lead to the inactivation of p53 tumor suppressor gene and promote UC-associated colorectal cancer progression^35^. In short, functional annotation and pathway enrichment analysis may provide new directions for the pathogenesis of UC.

In this study, six potential hub genes (*CXCL9, CXCL10, MMP-9, CHI3L1, CXCR2* and *S100A9*) in UC were identified. *CXCL9* and *CXCL10* genes also play important roles in tumors, such as melanoma and colorectal cancer^36,37^. As the expression pattern in tumor, the *CXCL9, -10, -11/CXCR3* axis impacts TAMs polarization. However, immune cells, show anti-tumor effect against cancer cells through paracrine *CXCL9, -10, -11/CXCR3* axis, the autocrine *CXCL9, -10, -11/CXCR3* signaling in cancer cells increases cancer cell proliferation, angiogenesis, and metastasis^38^. Studies have proved that the expression levels of *CXCL10* and *CXCL9* in tumor may be correlated with a poor prognosis of overall survival^39^. However, some studies also show that *CXCL10* and *CXCL9* may promote colonic tumorigenesis via promotes the cytokine-mediated mucosal injury and inflammation response^40^. In UC, *CXCL10* and *CXCL9* can recruit leukocytes to the site of inflammation and promote the occurrence and development of inflammation through *CXCL9, CXCL10, CXCL11/CXCR3* axis, which works primarily for immune cell migration, differentiation, and activation. *CXCL10* and *CXCL9* drive increased transcription of T-bet and RORγ, leading to Th1 polarization. After polarization, Th1 cells induce activation of CTLs, NK cells, and NKT cells through IFN-γ^41^.

In the analysis of the whole blood samples from UC patients, strikingly, the sensitivity of *MMP-9* and *S100A9* in the diagnosis of active UC was significantly higher than that of CRP, a commonly used clinical diagnostic molecule. This suggests that *MMP-9* and *S100A9* can be used as specific, sensitive and less invasive biomarkers of active UC, which is consistent with other reports^42,43^. A large number of studies have shown that *MMP-9* is highly expressed in the intestinal tissues of patients with UC and actively participated in the pathophysiological process of UC^44–46^. *MMP-9* can affect the tight connection between mucosal cells, increase intestinal mucosal permeability and aggravate mucosal barrier function^47^. Studies on *MMP-9* deficient mice also suggest that *MMP-9* is associated with mucosal damage in the early stages of colitis^48^. However, it has also been reported that *MMP-9* restricts the accumulation of reactive oxygen species and DNA damage in colon, and thus inhibits the occurrence colitis-associated cancer^49^. Therefore, understanding the role of *MMP-9* in UC and colitis-associated cancer is of great importance for exploring the treatment of UC and colitis-associated cancer.

*S100A9* is a calcium-binding protein mainly expressed by neutrophils, monocytes, and macrophages, and play a key role in the pathophysiology of various inflammatory diseases^50^. In rheumatoid arthritis, *S100A9*-mediated neutrophil migration and secretion of pro-inflammatory cytokines from monocytes lead to joint inflammation and joint destruction^51^. In gout, it`s a key factor that *S100A9* drives the production of sodium urate crystals and further induces the secretion of *IL-1β* in pathogenesis^52^. In contrast, in streptococcal pneumonia, blocking *S100A9* significantly inhibited the migration of neutrophils and macrophages to alveoli^53^. However, it remains unknown about the biological function of *S100A9* in intestinal inflammation. Our analysis indicated that the *IL-2/STAT5*, *IL6/JAK/STAT3* and *TNF‐α/NF‐κB* pathway were significantly activated in UC with higher expression of *S100A9*. This partly reveals the specific mechanism of *S100A9* in UC inflammatory response. In addition, angiogenesis and EMT pathways were also significantly activated in patients with high *S100A9* expression, suggesting that *S100A9* may be involved in UC-related tumor development.

In our animal model, *S100a9* mRNA and protein levels were significantly elevated in mice with colitis. Moreover, the expression of *S100a9* was significantly correlated with neutrophils and macrophages, which were closely related to the pathogenesis of UC. Inhibition of *S100a9* expression significantly reduced intestinal immune cell infiltration and inflammatory response in mice with colitis. Our results suggest that blocking *S100a9* inhibits inflammatory symptoms associated with UC and *S100a9* may be a potential therapeutic target for UC. However, there were some limitations in this study such as small sample size and lack of own sequencing data. Thus, more animal and clinical studies are needed to validate the results of this study in order to develop new treatments for UC in the future.

## Conclusions

In this study, through multiple bioinformatics approaches, immune cell infiltration characteristics were in active UC, and six genes (*CXCL9, CXCL10, MMP-9, CHI3L1, CXCR2* and *S100A9*) were screened out and verified as potential key genes of UC. Furthermore, *S100A9* serve as candidate diagnostic and therapeutic biomarkers for UC.

### Supplementary Information

Supplementary Information.

## Data Availability

The datasets used and analyzed during the current study are available from the corresponding author on reasonable request. The accession number for GEO are GSE87466, GSE107499, GSE59071 and GSE126124.
