# Supplementary material for: Identified S100A9 as a target for diagnosis and treatment of ulcerative colitis by bioinformatics analysis
Source: Sci Rep. 2024 Mar 6;14:5517. doi: 10.1038/s41598-024-55944-3 (PMC10917761; doi:10.1038/s41598-024-55944-3)
Supplement: Supplementary file 1 — Supplementary Information. [file 41598_2024_55944_MOESM1_ESM.docx]

**Identified *S100A9* as a target for diagnosis and treatment of ulcerative colitis by bioinformatics analysis**

Lulu Tan ^1,#^, Xin Li ^2,#^, Hong Qin ^1,#^,Qingqing Zhang^3^, Jinfeng Wang ^1^, Tao Chen ^1^, Chengwu Zhang ^1^, Xiaoying Zhang ^1,*^, Yuyan Tan ^1,*^

^1^ The First College of Clinical Medical Science, China Three Gorges University & Yichang Central People’ Hospital, Yichang, 443000, China;

^2^ Wuhan Asia Heart Hospital, Wuhan, 430022, China;

^3^ Haiyan County Hospital of Traditional Chinese Medicine, JiaXing, 314399, China.

^#^ These authors contributed equally to this work.

**Abstract**

Ulcerative colitis (UC) is a chronic, recurrent inflammatory bowel disease. UC confronts with severe challenges including the unclear pathogenesis and lack of specific diagnostic markers, demanding for identifying predictive biomarkers for UC diagnosis and treatment. We perform immune infiltration and weighted gene co-expression network analysis on gene expression profiles of active UC, inactive UC, and normal controls to identify UC related immune cell and hub genes. Neutrophils, M1 macrophages, activated dendritic cells, and activated mast cells are significantly enriched in active UC. *MMP-9, CHI3L1, CXCL9, CXCL10, CXCR2* and *S100A9* are identified as hub genes in active UC. Specifically, *S100A9* is significantly overexpressed in mice with colitis. The receiver operating characteristic curve demonstrates the excellent performance of *S100A9* expression in diagnosing active UC. Inhibition of *S100A9* expression reduces DSS-induced colonic inflammation. Theses identified biomarkers associated with activity in UC patients enlighten the new insights of UC diagnosis and treatment.

**Plain Language Summary**

⦁ *MMP-9, CHI3L1, CXCL9, CXCL10, CXCR2* and *S100A9* were identified as hub genes in active UC.

⦁ *S100A9* can be served as a potential diagnostic marker for UC.

⦁ *S100A9* is proven to be a potential therapeutic target of colitis.

**Keywords**:

ulcerative colitis, bioinformatics analysis, immune infiltration, diagnostic biomarkers, *S100A9*

^*^ **Corresponding author**

*CONTACT:* Yuyan Tan tyytyz@sina.com. The First College of Clinical Medical Science, China Three Gorges University & Yichang Central People’ Hospital, Yichang, 443000, China. Xiaoying Zhang z15623636995@126.com. The First College of Clinical Medical Science, China Three Gorges University & Yichang Central People’ Hospital, Yichang, 443000, China.

**Table. S1.** List of the primer sequences

| Primer name | Primer sequence (from 5’ to 3’) |
| --- | --- |
| M *S100a9* F | ACCACCATCATCGACACCTTC |
| M *S100a9* R | AAAGGTTGCCAACTGTGCTTC |
| M *Cxcl9* F | GCACGATCCACTACAAATCCC |
| M *Cxcl9* R | GGTTTGATCTCCGTTCTTCAGT |
| M *Cxcl10* F | CCAAGTGCTGCCGTCATTTTC |
| M *Cxcl10* R | TCCCTATGGCCCTCATTCTCA |
| M *Cxcr2* F | TGTCTGGGCTGCATCTAAAGT |
| M *Cxcr2* R | AGGTAACCTCCTTCACGTATGAG |
| M *Chi3l1* F | GTACAAGCTGGTCTGCTACT |
| M *Chi3l1* R | GTTGGAGGCAATCTCGGAAA |
| M *Mmp-9* F | GCAGAGGCATACTTGTACCG |
| M *Mmp-9* R | TGATGTTATGATGGTCCCACTTG |
| M *Il-6* F | ACCGCTATGAAGTTCCTCTC |
| M *Il-6* R | GTATCCTCTGTGAAGTCTCCT |
| M *Tnf-α* F | CTCACACTCACAAACCACCA |
| M *Tnf-α* R | TTGTCCCTTGAAGAGAACCT |
| M *β-actin* F | GTGGGCCGCTCTAGGCACCA |
| M *β-actin* R | CGGTTGGCCTTAGGGTTCAGGGG |


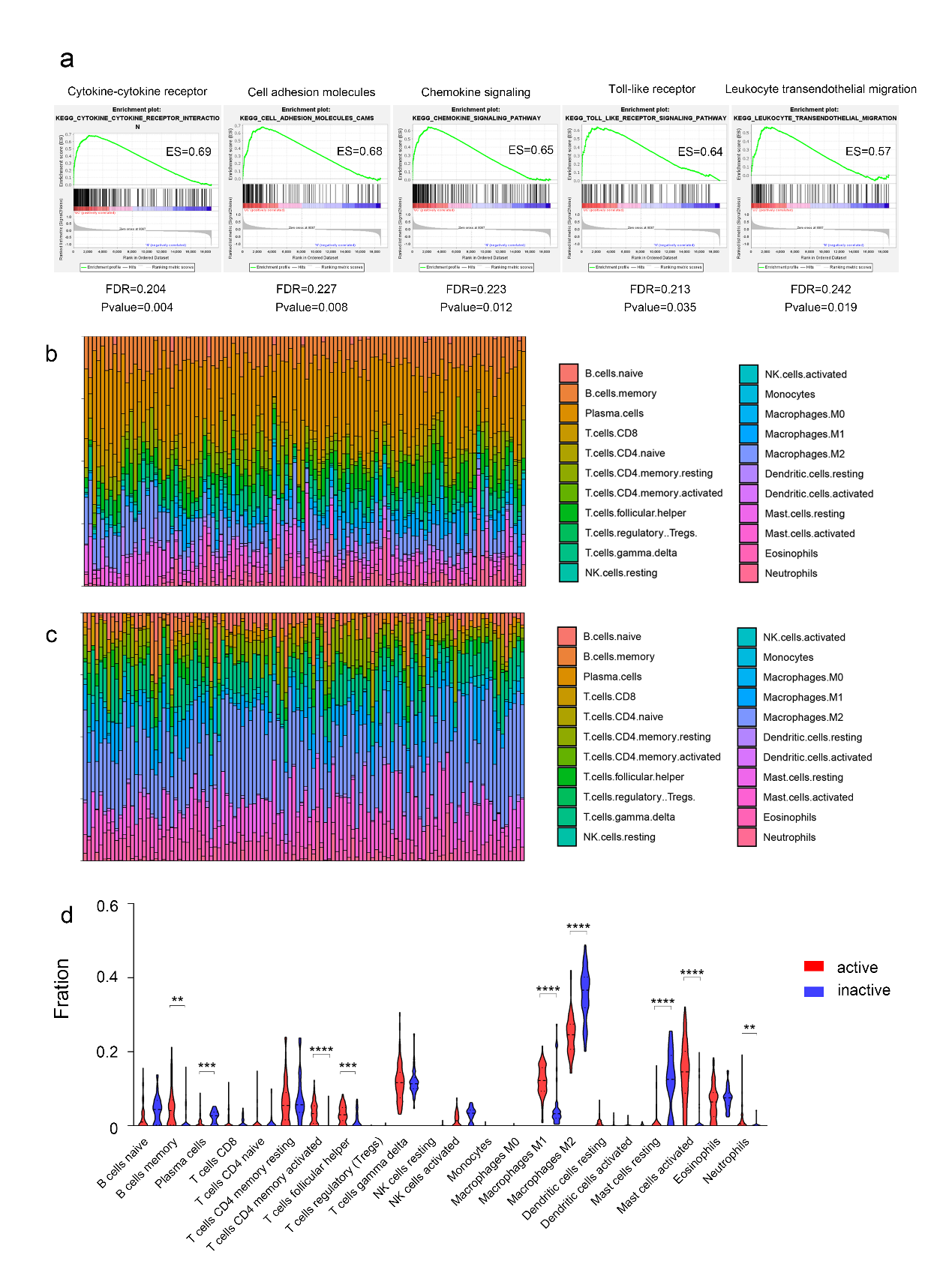


**Figure S1.** (a) GSEA of KEGG pathways in active UC and normal tissues. (b) Bar plot for the 22 immune cell subsets between 87 active UC and 21 normal tissues. (c) Bar plot for the 22 immune cell subsets between 75 active UC and 44 inactive UC samples. (d) Violin plot comparing the proportions of 22 types of immune cells between active UC and inactive UC.

**
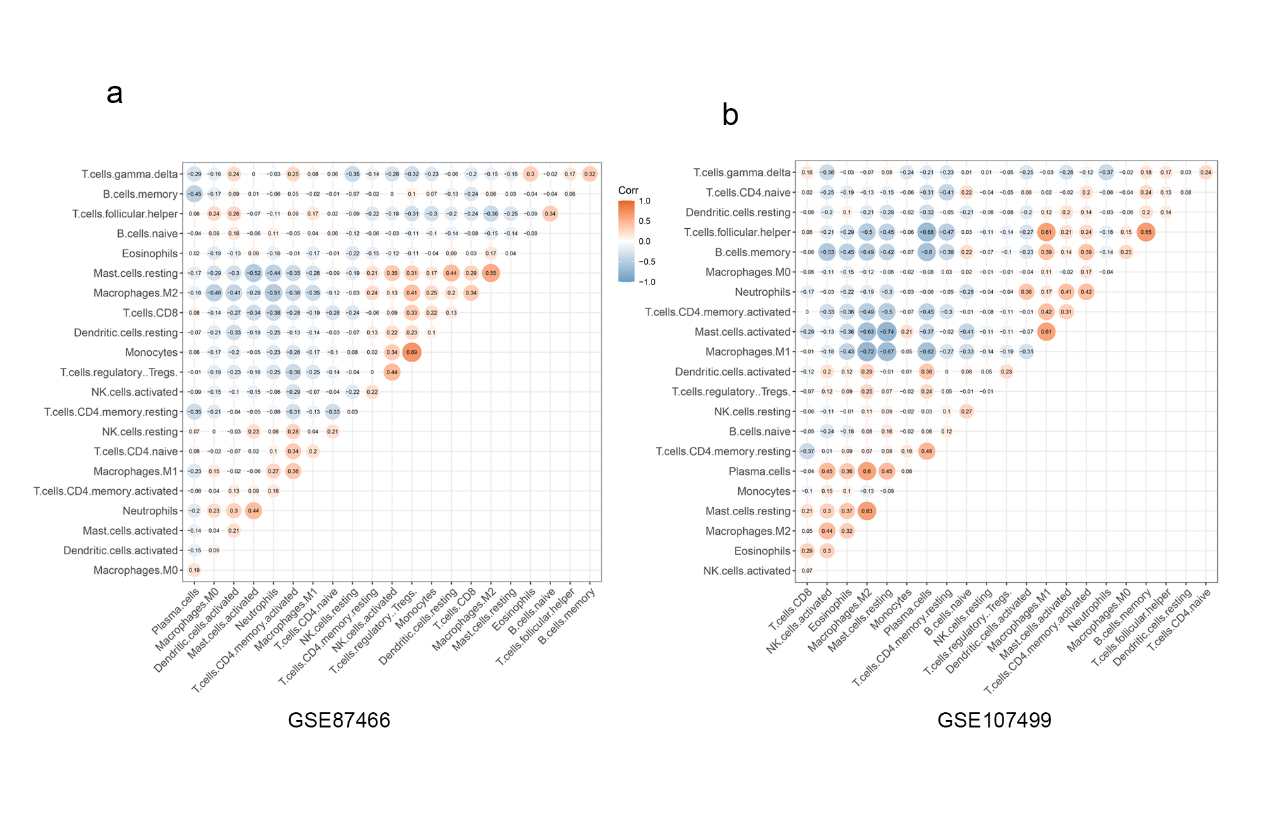
**

**Figure S2.** Pearson correlation analysis of 22 types of immune cells.


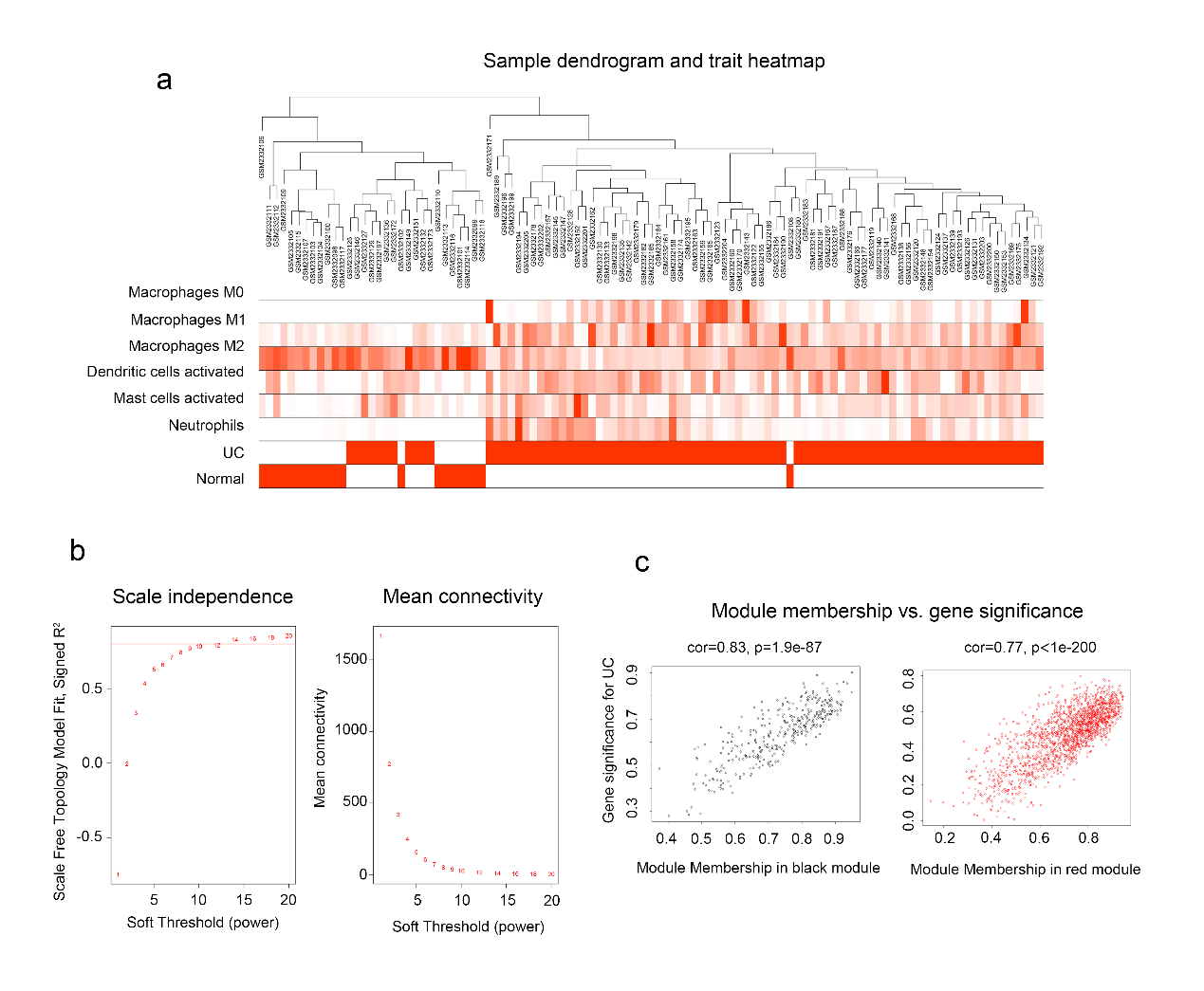


**Figure S3.** (a) Clustering dendrogram of 107 samples from WGCNA. One outlier sample was removed. (b) Determination of the soft threshold in the WGCNA algorithm. The soft-thresholding power was set to 12. (c) Identification of hub genes of black and red module by MM and GS filtering.


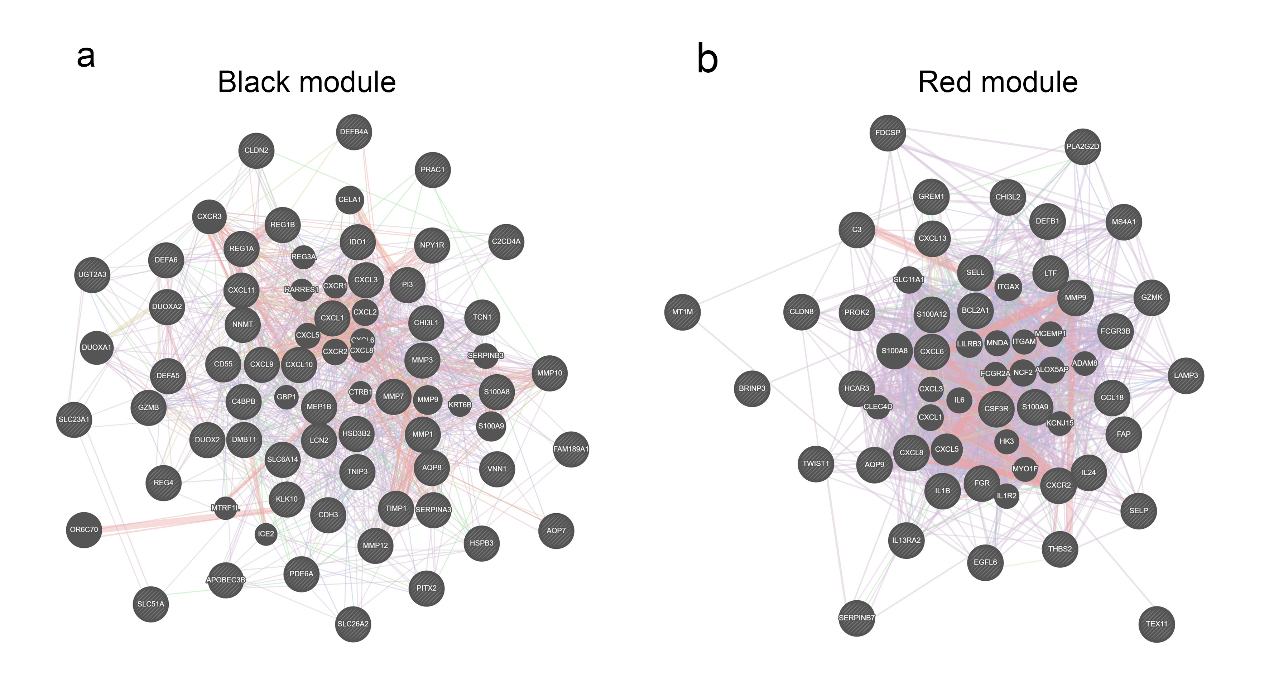


**Figure S4.** Images from [http://www.genemania.org](http://www.genemania.org/). PPI network for DEGs in the black (a) and red (b) modules.


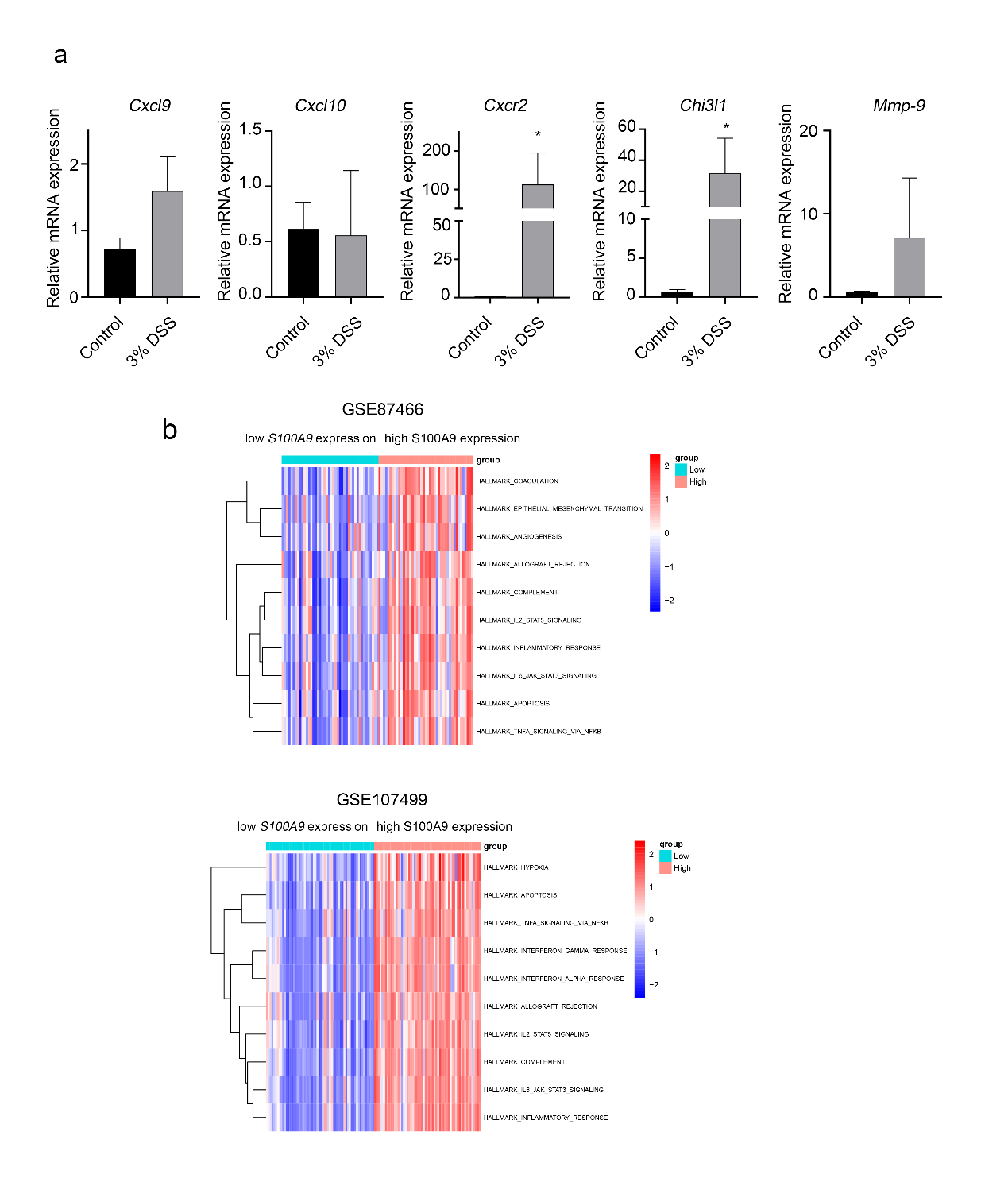


**Figure S5.** (a) The mRNA expression levels of *Cxcl9, Cxcl10, Cxcr2, Chi3l1* and *Mmp-9* in DSS-induced colitis mice and control. (b) Heatmap of enrichment score of hallmark gene sets between low‐ and high‐*S100A9* expression group in active UC patients.


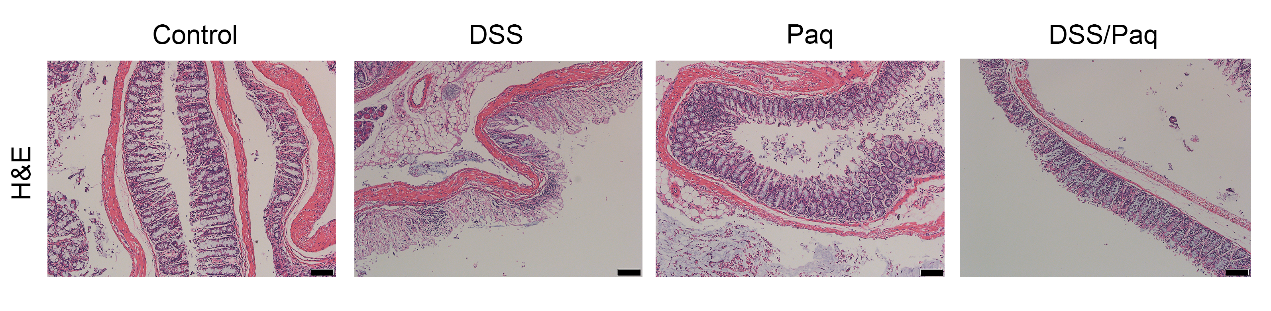


**Figure S6.** H&E staining analysis of colon tissues, scale bar: 100 µm.


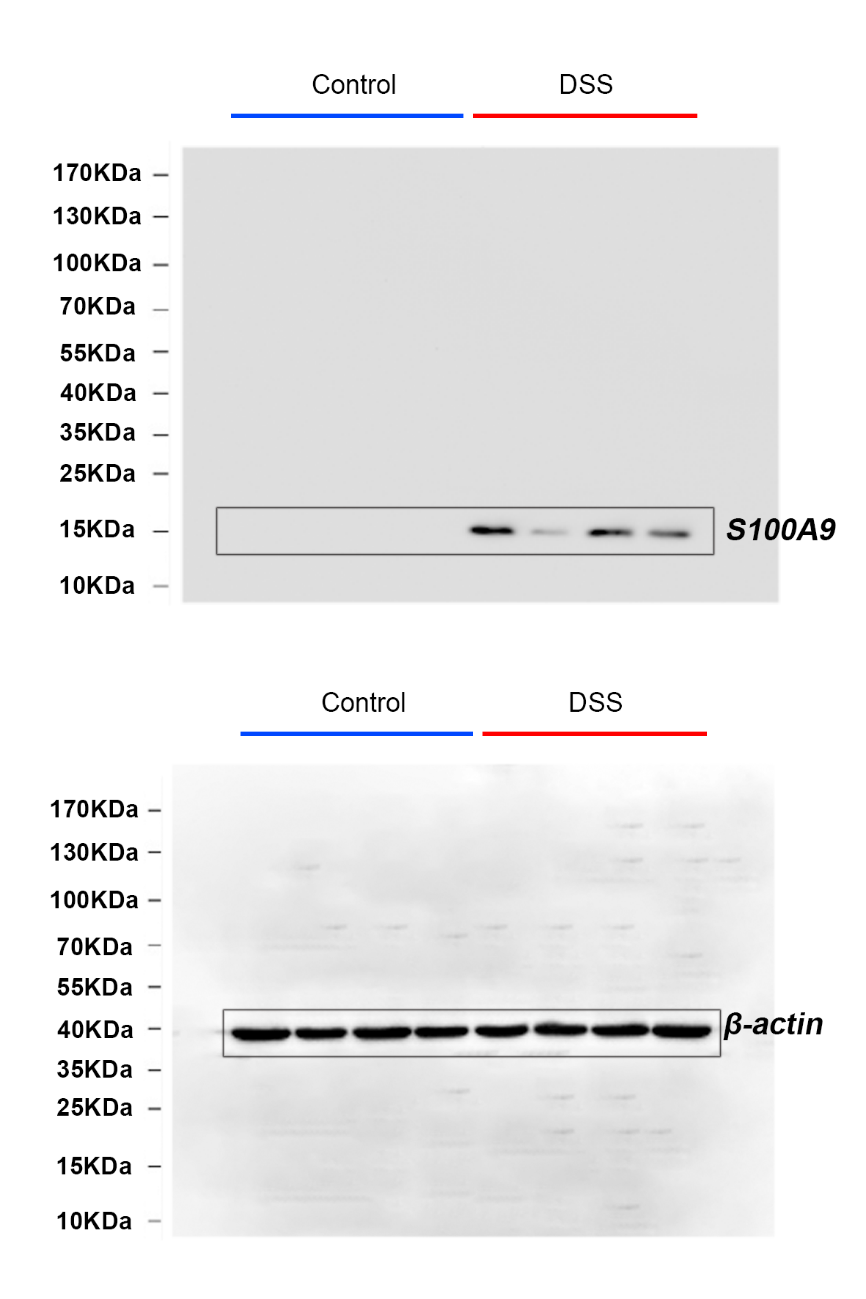


**Figure S7.** Original western blot images.
